# Comparison of rumen contents’ characteristics in Nguni and Bonsmara cows raised under two different grazing systems

**DOI:** 10.5455/javar.2024.k783

**Published:** 2024-06-08

**Authors:** Denis Kayima, Mhlangabezi Slayi, Ishmael Festus Jaja, Cletos Mapiye, Kennedy Dzama

**Affiliations:** 1 Livestock & Pasture Science Department, Faculty of Science & Agriculture, University of Fort Hare, Alice, South Africa; 2 Risk and Vulnerability Science Centre, University of Fort Hare, Alice, South Africa; 3 Department of Animal Sciences, Stellenbosch University, Stellenbosch, South Africa

**Keywords:** Ammonia, cattle breeds, grazing systems, rumen contents, volatile fatty acids

## Abstract

**Objective:**

This study aimed to evaluate rumen fermentation parameters influenced by both grazing system and breed.

**Materials and Methods:**

A 2 × 2 factorial design was employed, involving 40 cows with matched age, parity, and physiological status. The cows were evenly divided between Bonsmara and Nguni breeds, as well as communal and commercial grazing systems. Rumen fluid samples were collected and analyzed for parameters including ammonia-nitrogen (NH3-N), pH, temperature, and volatile fatty acids (VFAs).

**Results:**

Nguni cows exhibited significantly higher ruminal NH3-N levels (*p < *0.05) compared to Bonsmara, ranging from 69.05 to 96.78 mg/l. Commercial grazing demonstrated significantly higher NH3-N concentrations (*p < *0.05) than communal grazing. Ruminal pH, temperature, total VFAs, and specific VFAs (Iso-butyrate, valeric, and iso-valeric) did not show significant differences (*p > *0.05). However, total VFAs were slightly lower in communal grazing (78.87 mmol/l) than in commercial grazing (89.80 mmol/l). Acetate, propionate, butyrate, and the acetate to propionate ratio did not display significant differences (*p > *0.05) between breeds but varied between grazing systems. Communal systems had higher acetate and acetate to propionate ratio (*p < *0.05), while commercial systems showed higher propionate and butyrate levels (*p < *0.05).

**Conclusion:**

Grazing conditions significantly influenced rumen fermentation parameters, irrespective of breed. Further research is necessary to explore the relationship between forage conditions, diversity, and rumen fermentation within different grazing systems.

## Introduction

Understanding the effectiveness of feed utilization in bovine species and its association with breed type requires a thorough investigation into ruminal parameters in relation to grazing systems. In Africa, where natural pastures constitute a significant portion of the livestock feed resource, there is notable variability in the quality and quantity of forage across different grazing systems and seasons [1,2]. Numerous studies have underscored the impact of grazing on rumen parameters, exemplified by Friesian × Ankole F1 crossbred steers subjected to 100% grazing, displaying lower levels of rumen NH3-N and total volatile fatty acids (VFAs) compared to supplement and feedlot systems [3,4]. Molar proportion analysis further revealed distinct VFA profiles, with higher acetate levels in 100% grazing steers and elevated propionate levels in supplemented and feedlot systems [5]. Evaluating microbial and VFAs profiles, rumen pH, and ammonia has been recognized as indicators of feed utilization efficiency in feedlot systems [6,7]. However, limited research exists on rumen fermentation characteristics in pasture-based grazing systems due to challenges associated with obtaining rumen fluid and monitoring animal performance.

VFAs and NH3-N play pivotal roles as substrates for microbial protein synthesis within the rumen, contributing substantially to metabolizable energy and bacterial nitrogen, respectively [8,9]. Despite their importance, prediction errors for ammonia and VFA concentrations remain relatively high, approximately 60% and 20%, respectively [10]. This is attributed, in part, to unexplored sources of variation related to environmental conditions, feeding management, microbial profiles, and breed-specific idiosyncrasies in different grazing systems. In the realm of grazing systems, commercial and traditional practices represent distinctive approaches to managing livestock nutrition [11]. Commercial grazing systems, characterized by larger-scale operations, implement meticulous pasture management and controlled feeding practices, aiming to optimize forage utilization. In contrast, traditional grazing systems, often associated with smaller-scale or subsistence farming, rely predominantly on natural forages, with limited supplementation [7].

Interestingly, there is a lack of available data regarding the impacts of breed, grazing system, individual cow variability, and the interactions between breed and feeding/grazing systems [3,12]. Both breed and diet exert influence on bacterial and methanogen communities within the rumen [9,13], underscoring the importance of examining ruminal parameters and microbial profiles across different breeds to devise customized feeding strategies aimed at mitigating methane emissions [1,14]. Specifically, local beef breeds such as Nguni and Bonsmara remain understudied in terms of their impact on rumen fermentation dynamics. To address this gap, a comprehensive study focusing on Nguni and Bonsmara cattle breeds was undertaken, aiming to discern the effects of the grazing environment and inherent breed characteristics on rumen fermentation profiles. By delving into the unique characteristics of these local beef breeds and assessing their interactions with various grazing systems, the research aims to contribute valuable insights for more targeted and breed-specific management strategies.

## Materials and Methods

### Site description

The research was conducted at Bathurst Research Station and Alice in the Eastern Cape Province, South Africa. Alice is situated within the Raymond Mhlaba Local Municipality at 26°85′S latitude and 32°8′E longitude. The area experiences a mean annual rainfall of 480 mm and a temperature of 18.7°C [15]. Vegetation in the region includes *Aristida congesta, Themeda triandra, Digitaria eriantha, Sporobolus fimbriatus, Cynodon dactylon, Eragrostis spp*., and *Sporobolus africanus grass species*, with dominant tree species such as *Scutia myrtina, Acacia karroo*, and *Maytenus polyacantha* [16]. Bathurst Research Station is located at 26°49′E longitude and 33°30′S latitude, with an elevation of 708 m above sea level. The station experiences a mean annual rainfall of 624 mm and a temperature range of 1°C–12°C in winter and 13°C–29°C in summer. The vegetation in Bathurst is characterized by tall-grown thickets, with dominant species including *Euphorbias, Succulent aloes*, and understory thick woody lianas such as *Rhoicissus, Capparis, Secamone, Aloe*, and shrubby succulents such as *Crassulacae* and *Asphodelaceae*, along with thorny shrubs. However, the moister slopes facing south support thorny thickets consisting mainly of low-grown evergreen trees such as *Euclea, Cussonia, Pappea, Ptaeroxylon, Hippobromus, and Schotia*, as well as shrubs such as *Gymnosporia, Putterlickia, Azima, and Carissa*. There is minimal presence of trees and succulent shrubs due to low radiation intensity, resulting in a poorly developed herbaceous layer [16].

### Experimental procedure

The investigation analyzed grazing systems, specifically communal and commercial, utilizing two distinct cattle breeds, namely Nguni and Bonsmara, within a 2 × 2 factorial design. Communal grazing involves shared land ownership with equal access, fostering community involvement and subsistence farming. Decision making is collaborative. Commercial grazing is profit-driven, with privately owned land and individual management control. It focuses on larger-scale operations for commercial production, distinguishing it from communal systems. For this study, 40 healthy cows within a consistent age range were chosen, with 10 cows representing each breed in both grazing systems. The two breeds under investigation, Nguni and Bonsmara, are distinct entities within the realm of cattle populations, each possessing unique genetic characteristics that contribute to their recognition and differentiation.

Nguni cattle are recognized as a distinct breed, not merely a population. Indigenous to Southern Africa, Nguni cattle have a rich history and are well-adapted to diverse environmental conditions [4]. They are esteemed for their hardiness, adaptability, and resistance to various diseases. Nguni cattle often exhibit a variety of coat colors and patterns, reflecting their genetic diversity. Their small to medium size, humpless appearance, and distinctive horns contribute to their recognizable features. Bonsmara cattle, like the Nguni, are also recognized as a distinct breed. Developed in South Africa through systematic crossbreeding, Bonsmara cattle are known for their adaptability, good maternal instincts, and desirable meat qualities. The breed was specifically developed to thrive in the harsh African environment, and its genetic makeup includes contributions from Afrikaner, Shorthorn, and Hereford cattle. Bonsmara cattle typically display a solid red coat color and possess a hump over their shoulders, distinguishing them from humpless breeds. The genetic distinctions between Nguni and Bonsmara cattle contribute to variations in their physical attributes, adaptability, and performance. While both breeds are well-adapted to African environments, Nguni cattle are characterized by greater genetic diversity, including a range of coat colors and patterns. In contrast, Bonsmara cattle are more uniform in appearance, displaying a consistent red coat color.

The cows were selected to ensure parity, physiological status, and unrestricted grazing access in the field. The stomach tube method was employed for sampling and fluid collection from each cow [17]. All animals were carefully and ethically handled as requested by the institution committee (MUC041SKAY01) to ensure their well-being throughout the sampling process. Before sample collection, animals were subjected to a brief period of anesthesia to minimize any potential stress or discomfort. We followed a standardized procedure for animal anesthesia, administering an appropriate dose of the established anesthetic agent isoflurane [18]. An experienced veterinarian conducted the anesthesia administration, closely monitoring the animals to maintain a controlled and stable anesthetic state. Subsequently, a flexible PVC tube, measuring 2 mm in thickness with an internal diameter of 6 mm and equipped with approximately 20 holes, each with a diameter of 3 mm, was utilized. This tube, affixed with a 12 cm long probe (Cristallo Extra, FITT S.p.A., Sandrigo, Italy), was inserted through the esophagus to a depth of approximately 120–150 cm, reaching the rumen. Rumen fluid (approximately 50 ml) was extracted using a vacuum pump (operating at about 7 bar: Vacuum brand MZ 2C, Wertheim, Germany). Rumen fluid pH was immediately measured using a pH-meter (Crison GLP 21, Barcelona, Spain). Immediately after collection, the sample container was capped tightly to prevent air exposure. The samples were stored in a pre-cooled container or cooler with ice packs to maintain the temperature near the cow’s body temperature (38°C–39°C). The samples were transported to the laboratory as quickly as possible. They were analyzed within a few hours of collection to minimize changes in microbial activity and composition.

### Fatty acid (FA) profile for pasture samples

Pasture samples were collected from the study site for analysis of FA profiles. Random and blended pasture samples were obtained using a quadrant measuring 1 **×** 1 m from a belt transect spanning 100 **×** 25 m at each paddock in both the communal and commercial grazing areas. These pasture samples were then dried in an oven for 48 h at 60°C. After drying, the samples were ground and sieved through a 2 mm sieve. From the dried and sieved pasture samples, 100 mg was extracted using 5 ml of n-hexane. The internal standard utilized was 100 µl of 0.1 ml/l of heptadecanoic acid (17:0) in n-hexane, added before the addition of 1 ml of 2.5% methanolic acid as a transmethylating reagent. Thermo TRACE 1300 series gas chromatography (Thermo Electron S.P.A, Strada Rivoltana, 200090 Rodana, Milan, Italy) was employed to analyze the fatty acid methyl esters (FAMEs), utilizing a flame ionization detector and a 30 m TR-FAME capillary column with internal diameter and film thickness of 0.25 mm and 0.25 µm, respectively (Cat. No. HY260M142P, Anatech, Cape Town, South Africa). The analysis was conducted over a run time of 40 min, with an injection volume of 1 μl. Gas chromatography conditions included an initial temperature of 50°C for 1 min, reaching a final temperature of 240°C. The injector/detector temperature settings were 240°C/250°C, with a hydrogen gas flow rate of 40 ml/min. The FAMEs for each sample were determined by comparing their retention times to those of the standard (Supelco™ 37 Component FA methyl esters mix, Cat no. CRM47885, Supelco, USA). Total FAs were quantified and expressed as gm/100 gm of total FAs, with analyses performed in quadruplicate.

### Filtering and preservation of rumen fluid samples

The rumen fluid samples underwent filtration through a four-layer cheesecloth into 50 ml tubes sourced from Merck KGaA, Darmstadt, Germany. Following filtration, the tubes were securely covered, placed on ice, and promptly transported to the laboratory. To maintain the integrity of the strained rumen fluid for subsequent Ammonia-N determinations, each sample received 1 ml of 20% concentrated H_2_SO_4_ added to 5 ml of the fluid, after which they were stored at −20°C. For VFA analysis utilizing GC, a 10 ml portion of each filtered sub-sample underwent acidification by the addition of 2.5 ml of 25% orthophosphoric acid (*w*/*v*) until a pH below 2 was achieved. The acidified samples were then stored at −20°C, following the methodology delineated by previous studies [19,20].

### Determination of the amount of ammonia and VFAs

Ammonia-nitrogen levels were determined using colorimetry, following the methodology outlined by McCracken et al. [20]. Gas chromatography was employed for measuring concentrations of VFAs, utilizing a Thermo Scientific™ TRACE™ 1300 instrument equipped with a Thermo TriPlus RSH Autosampler. The analysis utilized a Phenomenex Zebron ZB-FFAP capillary GC column with specifications according to Noel et al. [11] (0.25 mm internal diameter, 30 m length, and 0.25 µm film thickness). Crotonic acid served as the internal standard, and 1 µl injections were made during the 18-min run. Thermo Scientific Xcalibur™ Software was used for calculating VFA concentrations, expressed as mmol/l. Individual VFAs were then converted to mmol/100 mmol of the total VFAs to ensure accurate representation.

### Statistical analysis

The collected data underwent analysis using the Statistical Analysis Software PROC MIXED of SAS version 9.4. The grazing system, breed, and their interactions were treated as fixed factors, with each individual cow considered an experimental unit. For the FA profile of pasture samples, the grazing system was the sole factor considered. Statistical analyses were performed on the FA profile data to determine significant differences between communal and commercial grazing systems. ANOVA was utilized to assess whether the means of the FA profiles differed between the two groups. Significance was determined using the least significant difference method, with means considered significantly different at *p < *0.05. The analysis adopted the following model:

*yijk*=*μ*+*τi*+*δj*+(τ*δ)*ij*+*εijk*

where:

*y_ijkh_*= response variable, i.e., VFAs, Ammonia, pH, and temperature*, μ *= the overall mean, τ*_i_*= the effect of grazing system where (*i = 2*; communal and commercial grazing systems), *δ_j_*= the effect of breed where (*_j _*= 2; Nguni and Bonsmara breeds), (τ*δ)*_ij_* = effect of interaction between grazing systems and breed and *ε_ijkh = _*random error.

## Results

### FA composition of pasture samples from grazing fields

The FA profile of pasture samples was significantly influenced by the grazing system employed (*p < *0.05; Table 1). α-linolenic acid (18:3n-3) emerged as the most abundant polyunsaturated fatty acid (PUFA) in both grazing systems, comprising 39.66 to 27.41 gm/100 gm of total FAs. Notably, pastures from commercial settings exhibited a significantly higher content of α-linolenic acid (*p < *0.05). Palmitic acid (16:0) was the second most prevalent FA, ranging from 23.23 to 22.12 gm/100 gm of total FAs, with consistent levels across both grazing systems. Linoleic acid (18:2n-6) followed as the subsequent major component, displaying higher levels (*p < *0.05) in the communal grazing system, ranging from 25.56 to 19.17 gm/100 gm of FAs. Total fat content was also significantly impacted by the grazing system, with commercial pastures showing higher levels (*p < *0.05) compared to those from communal grazing fields.

In terms of saturated fatty acids (SFAs), communal grazing pastures exhibited a higher (*p < *0.05) percentage of 14:0 and 15:0, although the total SFA content did not significantly differ between the grazing systems (Table 1). The proportions of 16:1, 17:1, 22:1, and total monounsaturated fatty acids (MUFAs) were higher (*p < *0.05) in communal grazing pastures, while the proportion of 18:1n-9 did not significantly differ (*p > *0.05) between the grazing systems. Total PUFAs and the proportion of 20:2 were higher (*p < *0.05; Table 1) in pastures from commercial settings. The ratio of PUFAs to SFAs (P:S) did not differ significantly among the grazing systems. However, the total n6 content was higher (*p < *0.05) in communal grazing pastures, while the total n3 content was higher in commercial grazing pastures. Additionally, the ratio of n6:n3 was higher (*p < *0.05; Table 1) in commercial grazing systems.

### Impact of breed and grazing systems on rumen fermentation parameters

The effects of grazing system, breed, and their interaction on various ruminal parameters, including VFAs production, ruminal ammonia concentration, pH, and temperature, are summarized in Table 2. Ruminal temperature and pH remained unaffected (*p > *0.05) by both the grazing system and breed, as well as their interaction. In contrast, ruminal ammonia concentration exhibited significant differences (*p < *0.05) among grazing systems and breeds. Nguni cows demonstrated higher (*p < *0.05) ruminal ammonia concentration compared to Bonsmara cows. Regardless of breed, commercial cows exhibited elevated (*p < *0.05) ruminal ammonia-nitrogen levels compared to their communal counterparts.

**Table 1.** Effect of grazing system on the different FA profiles of pasture samples.

FA	Commercial	Communal	SEM	*p-*value
14:0	0.49	0.61	0.031	0.016
15:0	0.17	0.30	0.036	0.025
16:0	22.12	23.23	0.424	0.086
18:0	2.06	2.30	0.155	0.283
20:0	5.04	4.50	0.588	0.529
22:0	1.04	1.13	0.144	0.663
∑SFA	30.92	32.07	0.806	0.328
16:1	0.17	0.40	0.064	0.021
17:1	0.17	0.40	0.064	0.021
18:1n-9	2.85	3.05	0.104	0.202
22:1	0.38	1.21	0.135	<0.0001
∑MUFA	3.57	5.06	0.261	0.001
20:2	0.26	1.02	0.087	<0.0001
18:2n-6	19.17	25.56	1.174	0.002
18:3n-3	39.66	27.41	1.141	<0.001
20:4n-6	1.98	4.71	0.530	0.003
20:5n-3	4.45	4.16	0.251	0.427
∑PUFA	65.51	62.87	0.725	0.022
P:S	2.12	1.98	0.073	0.204
∑FA in ug/gm	13017.00	8449.40	789.800	0.001
∑n-6	21.15	30.33	1.457	0.001
∑n-3	44.10	31.57	1.180	0.0001
n-6:n-3	0.48	0.99	0.033	0.001

FA = Fatty acids, SFA = Saturated Fatty acids, MUFA = Monounsaturated fatty acids, PUFA = Polyunsaturated fatty acids, n-6 = Omega 6, n-3 = Omega 3, P:S = Polyunsaturated fatty acids to Saturated fatty acids, ratio n-6: n-3 = omega 6 to omega 3 ratio, ug/gm = Microgram per gram.

**Table 2.** Means of rumen fermentation parameter as effected by breed, grazing system and their interaction.

Parameter	Breed			razing system			*p-*value		
	Bonsmara	Nguni	SEM	Communal	Commercial	SEM	Breed	Grazing system	Breed*Grazing system
Ammonia- N (mg/l)	69.05^b^	96.78^a^	6.613	73.43^b^	92.41^a^	6.613	0.005	0.050	0.935
pH	6.85^a^	6.84	0.048	6.87	6.81	0.048	0.890	0.330	0.753
Temperature (°C)	34.21	34.18	0.082	34.08	34.31^a^	0.082	0.785	0.055	0.838
Total VFAs (mmol/l)	85.68	87.65	5.871	78.87	89.80^a^	4.793	0.791	0.133	0.132
Individual VFAs (mmol/100 mmol)									
Acetate	65.55^a^	64.26^a^	1.598	67.83^a^	59.90^b^	1.305	0.275	0.001	0.460
Propionate	17.33^a^	19.90^a^	0.953	17.73^b^	20.65^a^	0.778	0.094	0.021	0.566
Butyrate	13.43^a^	11.94^a^	1.006	10.70^b^	15.65^a^	0.821	0.322	0.001	0.914
Iso-butyrate	1.19	1.03	0.253	0.94	1.31	0.207	0.892	0.230	0.303
Valerate	1.55	1.80	0.181	1.75	1.56	0.148	0.609	0.371	0.253
Iso-valerate	0.94	1.06	0.086	1.06	0.93	1.06	0.597	0.218	0.226
Acetate: Propionate	3.83^a^	3.33^a^	0.188	3.88^a^	2.97^b^	0.154	0.051	0.001	0.284

VFAs = Volatile fatty acids, Ammonia-N = Ammonia- Nitrogen.

^a–b^Different letters in the same row are significantly different (*p* ≤ 0.05). SEM, Standard Error of the Mean.

Total ruminal VFAs and the proportions of iso-butyrate, valerate, and iso-valerate showed no significant variations (*p > *0.05) between breeds, regardless of the grazing system. Similarly, no significant differences were observed between grazing systems, irrespective of breed. However, the total VFA concentration slightly decreased in communal grazing systems (78.87 mmol/l) compared to commercial grazing systems (89.80 mmol/l). Acetate, propionate, butyrate proportions, and the acetate to propionate ratio exhibited significant distinctions (*p < *0.05) between grazing systems while remaining comparable (*p > *0.05) among breeds. Cows within communal grazing systems displayed higher acetate concentration (*p < *0.05) at 67.83 and an elevated acetate-to-propionate ratio (*p < *0.05) at 3.88. Conversely, propionate (*p < *0.05) and butyrate (*p < *0.05) proportions were higher in cows from commercial grazing systems.

## Discussion

This study conducted an extensive exploration into the intricate relationships among distinct grazing systems, breed types, and their impact on rumen fermentation patterns and the ruminal environment. Our investigation yielded valuable insights into the collective influence of these factors, particularly emphasizing their role in shaping ruminal ammonia-nitrogen concentration—a critical parameter indicative of feed utilization efficiency and microbial activity [13]. Both grazing systems and breed types were found to distinctly influence ruminal ammonia-nitrogen concentration, providing significant implications for ruminant management and nutrition. Notably, our findings highlighted that commercial grazing systems and the Nguni breed were associated with elevated levels of ruminal ammonia-nitrogen concentration. This observation aligns with established thresholds in previous studies [21–23], emphasizing the sensitivity of this parameter to the multifaceted interplay between grazing practices and genetic backgrounds. The elevated levels observed in commercial systems prompt further exploration into potential contributing factors. The study suggests that diet composition, feed management practices, and intensified production systems in commercial grazing may play crucial roles in influencing rumen fermentation parameters. The nuanced understanding of these factors’ collective influence on rumen physiology is crucial for refining ruminant management strategies in commercial settings and advancing our broader comprehension of rumen fermentation dynamics.

Similarly, the elevated ruminal ammonia-nitrogen concentration observed in the Nguni breed could stem from inherent genetic traits impacting nitrogen metabolism or dietary preferences. This finding resonates with existing literature, emphasizing the intricate relationship between ruminal ammonia-nitrogen concentration and ruminant nutrition [8,24,25]. The interdependence of grazing systems and breed types in shaping this crucial parameter warrants prudent consideration in designing strategies to enhance feed efficiency, animal health, and overall performance. The study delves further into the potential role of specific bacterial populations, such as *Clostridium aminophilum, Peptostreptococcus anaerobius*, and *Clostridium sticklandii*, known for their association with elevated ammonia-nitrogen concentrations [23,26,27]. The interplay between these bacterial populations, host genetics, and the varying quality of pasture in distinct grazing systems contributes to the intricate tapestry of ruminal dynamics. The study broadens our understanding of the complex relationships within the rumen ecosystem, shedding light on microbial activity and nutrient utilization. Interestingly, ruminal temperature and pH remained consistent across grazing systems and breed types, mirroring optimal conditions reported in similar studies involving grazing animals. Although the total VFAs concentration showed no significant changes among breeds and grazing systems, a relatively lower level was observed. This observation may be attributed to the study’s execution during the dry season, characterized by more recalcitrant plant matter hindering microbial degradation. Distinct proportions of VFAs further underscored the dynamic responses within the rumen ecosystem. Differences in acetate, propionate, and butyrate proportions between grazing systems revealed an intricate balance influenced by the composition of pastures. These variations can be ascribed to the differential fat and FA profiles of pastures in different grazing systems, impacting fermentation patterns [28–30].

In summary, this study’s outcomes emphasize the intricate interplay between grazing systems, breed genetics, and ruminal fermentation patterns. Commercial grazing systems fostered propionate and butyrate production, while communal grazing systems promoted acetate production. These findings contribute to the broader understanding of livestock production optimization, considering the intricate relationships between grazing strategies, breed genetics, and rumen fermentation dynamics [31,32]. Moreover, the study underscores the need for holistic management strategies that harness these factors to enhance livestock productivity and overall well-being.

## Conclusion

Our study highlights the significance of grazing systems and cow breeds in modulating rumen fermentation patterns and the ruminal environment, as exemplified by the notable differences in ruminal ammonia-nitrogen concentration. The discerned associations between commercial grazing systems, the Nguni breed, and elevated ruminal ammonia-nitrogen levels provide a foundation for targeted interventions aimed at optimizing ruminant nutrition and management practices. However, further investigations are warranted to unravel the underlying mechanistic interactions that give rise to these observations, thereby contributing to a holistic understanding of ruminant digestive physiology and its implications for sustainable livestock production.

## List of abbreviation

%, percentage; mg/l, milligrams per liter; ^o^C, degrees Celsius; mmol/l, millimoles per liter; ug/gm, Microgram per gram; FA, Fatty acids; MUFA, Monounsaturated fatty acids; P:S, Polyunsaturated fatty acids to Saturated fatty acids; PUFA, Polyunsaturated fatty acids; SFA, Saturated Fatty acids; VFA, Volatile Fatty Acids.
